# Editorial: Genomics of pathogens and vectors

**DOI:** 10.3389/fgene.2024.1483676

**Published:** 2024-09-12

**Authors:** Gabriel Luz Wallau, Augusto Simoes-Barbosa, Túlio de Lima Campos

**Affiliations:** ^1^ Núcleo de Bioinformática, Instituto Aggeu Magalhães (IAM), Fundação Oswaldo Cruz (Fiocruz/PE), Recife, Brazil; ^2^ Departamento de Entomologia, Instituto Aggeu Magalhães (IAM), Fundação Oswaldo Cruz (Fiocruz/PE), Recife, Brazil; ^3^ Department of Arbovirology, Bernhard Nocht Institute for Tropical Medicine, WHO Collaborating Center for Arbovirus and Hemorrhagic Fever Reference and Research, National Reference Center for Tropical Infectious Diseases, Hamburg, Germany; ^4^ School of Biological Sciences, Faculty of Science, University of Auckland, Auckland, New Zealand

**Keywords:** genomic surveillance, genomes, pathogens, antimicrobial resistance, virulence factors, diagnostics

## Introduction

Advances in high throughput genomic sequencing and bioinformatics have revolutionized our understanding of the biology of pathogenic organisms, including those transmitted by vectors. The use of these technologies has enabled precise identification and tracking of pathogens based on mutational profiles. Elucidating transmission dynamics and evolutionary patterns is crucial for informing public health decision making, and this has become even more critical in our modern times as the accelerated climate change is rapidly altering pathogen transmission dynamics. Genomics has also been crucial to monitor antimicrobial resistance genes and other molecular aspects that can impair treatment efficacy. Furthermore, these methods have uncovered virulence factors, providing insights into mechanisms that enable pathogens to disperse in the environment, invade their hosts and cause disease. The information revealed by pathogen genomics is also pivotal for developing accurate diagnostics, effective vaccines, and targeted therapeutics thus becoming indispensable for advancing public health strategies aimed at tackling infectious diseases. With this in mind, the thematic issue “*Genomics of Pathogens and Vectors*” highlighted the use of genomics and bioinformatics to investigate a diversity of established and emerging pathogens. Together, these articles showcase how the application of these technologies contributes to advancing our knowledge on pathogen dispersion and transmission, resistance to treatment, virulence and pathogenicity, as well as diagnostics.

## Enhancing molecular epidemiology

Genomics enables and enhances molecular epidemiology by providing detailed insights into the genetic factors that influence disease patterns and distribution within populations. For example, Chem et al. analysed genetic data from dengue viruses collected in Malaysia from 2015 to 2021. They found a high diversity of dengue viruses and frequent turnover of different strains, suggesting extensive movement of the virus between countries and regions. Wang et al. identified mutations in key genes associated with increased transmission of *Mycobacterium tuberculosis*, the causative agent of tuberculosis and one of the deadliest infectious disease worldwide. Focusing on population genomics of *Klebsiella pneumoniae* from China, Feng et al. showed a high prevalence of strains resistant to multiple antibiotics that may undergo rapid spread. This is a concerning bacterial pathogen in hospital settings, presenting a mortality rate of up to 40% in bloodstream infections. By sequencing the genomes of *Burkholderia pseudomallei* from environmental samples in Ghana, Schully et al. provide a better understanding on the distribution of this emerging pathogenic bacterium that causes meliodosis, a serious but neglected illness.

## Monitoring antimicrobial resistance genes and virulence factors

Revealing genes that trigger antimicrobial resistance and virulence factors is crucial for understanding and combating infectious diseases. To understand the genetic relatedness between growth rate and disease severity, Zhu et al. compared the genomes of different *Mycobacterium* species and found that loss of virulence factors is central to this relationship. Following a similar comparative genomics approach, Flores-Oropeza et al. found a variety of genes associated with antibiotic resistance in *Escherichia coli* isolated from women with recurrent urinary tract infections. The researchers also identified a variety of genetic factors and phenotypic variations implicated on this disease. Another study by Cai et al. compared the genomes of the emerging human pathogen *Paraclostridium bifermentans* to a related bacterium species *Paeniclostridium sordellii*, revealing that the former has a larger and more plastic genome than expected and may encode more virulence factors than the reference species.

Studies in commensal and environmental species were also featured. In one example, Chen et al. analysed the genomes of *Cutibacterium granulosum*, a bacterium found on human skin. They found a high diversity of strains and identified genes potentially linked to antibiotic resistance and molecules linked to virulence. Yuan et al. analysed the genomes of *Ralstonia picketti*i, a bacterium found in environmental soil and water which can opportunistically cause human infections. These authors described a flexible genome that allows adaptation to different environments as well as antimicrobial resistance genes. Xiao et al. sequenced the genome of *Ralstonia solanacearum*, a bacterium causing a wilt disease in tobacco. They found high similarity across strains but also unique features on the phytopathogenic strain which may help explain how the latter infects tobacco and other plants. Lastly, Mpeyako et al. analysed genomes of *Trichomonas tenax* and compared specific gene sets between this species and *Trichomonas vaginalis,* two mucosal-dwelling protozoans of humans. While the latter is recognized as a true urogenital pathogen, the former has only been linked to gum disease. The authors identified genes linked to virulence across the two species that are involved on interactions with host cells and mucosal microbiota, supporting the notion that *T. tenax* may be a direct causative agent of gum disease.

## Advancing molecular diagnostics

The integration of cutting-edge genomic and bioinformatic technologies enable timely detection of pathogens with improved precision and sensitivity. In this context, Yao et al. assessed the value of shotgun metagenomics for diagnosing respiratory tract infections. The authors showed that the metagenomic approach was more sensitive and accurate than traditional methods that rely on hypothesis-driven detection or pathogen isolation, especially for diagnosing acute infections. To overcome issues with growth and isolation of the fastidious pathogen *Francisella tularensis*, Isidro et al. developed a new method to capture and sequence its genome directly from animal samples. They successfully sequenced many full genomes, including mixed genotypes from single samples. This method will be useful for studying the spread of this zoonotic bacterium in wildlife and may be applicable to other pathogens and settings where microbial isolation is technically challenging.

## Conclusion

In summary, the Research Topic of articles selected for this thematic issue showcases the transformative potential of genomics in advancing our battle against infectious diseases. Evolution, emergence and re-emergence of pathogens are reported with the increasing threats of antimicrobial resistance and hypervirulence. Meanwhile, recent and ongoing anthropogenic impact is changing the dynamics of emerging and reemerging pathogen transmission across the globe. Together, this underscores the urgency for expanding research, development and application of genomics and related technologies to safeguard public health. Genomics serves as a powerful tool for pathogen surveillance, diagnosis, and development of effective countermeasures. Equitable access to these technologies will strengthen global health security while fostering scientific growth, collaboration and innovation.

